# The evolution of monogamy in response to partner scarcity

**DOI:** 10.1038/srep32472

**Published:** 2016-09-07

**Authors:** Ryan Schacht, Adrian V. Bell

**Affiliations:** 1 Department of Anthropology, University of Utah 270S 1400 E, Salt Lake City, UT 84112, USA

## Abstract

The evolution of monogamy and paternal care in humans is often argued to have resulted from the needs of our expensive offspring. Recent research challenges this claim, however, contending that promiscuous male competitors and the risk of cuckoldry limit the scope for the evolution of male investment. So how did monogamy first evolve? Links between mating strategies and partner availability may offer resolution. While studies of sex roles commonly assume that optimal mating rates for males are higher, fitness payoffs to monogamy and the maintenance of a single partner can be greater when partners are rare. Thus, partner availability is increasingly recognized as a key variable structuring mating behavior. To apply these recent insights to human evolution, we model three male strategies – multiple mating, mate guarding and paternal care – in response to partner availability. Under assumed ancestral human conditions, we find that male mate guarding, rather than paternal care, drives the evolution of monogamy, as it secures a partner and ensures paternity certainty in the face of more promiscuous competitors. Accordingly, we argue that while paternal investment may be common across human societies, current patterns should not be confused with the reason pairing first evolved.

## Introduction

The evolution of monogamy in humans is commonly argued to have been driven by the need for paternal investment (reviewed in ref. 1). Specifically, the unique suite of human life history traits - altricial young, a long juvenile period, delayed maturity, extension of the lifespan and large brains - are often claimed to have resulted from the emergence of male care^2^. While these claims are contentious for various reasons^3^,^4^, ultimately they ignore the social dilemma males face regarding shifting reproductive strategies^5^. The switch from a mating effort intensive strategy (i.e., multiple mate appropriation) to a paternal investment strategy (i.e., resource production) is prone to free-riding by male competitors, raising questions about a straightforward promiscuity-to-monogamy mating system transition in human evolution^1^,^6^. A commonly ignored pathway, which may provide some degree of resolution to this puzzle, is through frequency dependent payoffs to mate guarding^7^,^8^. Male mate guarding is present across animal taxa and is often expressed as monogamy without paternal care^9^.

### Why do men care?

Human offspring are born highly vulnerable and slow-maturing and generally receive investment from both parents^2^,^10^. However, biparental care is unusual across mammals^11^, likely because it does not result from a simple cooperative system of joint investment by males and females. While each investment in an offspring provides benefits to the parent, it also benefits his/her partner, creating conflict between parents over who pays the ‘costs’ of care (e.g., reduced mating opportunities^12^,^13^,^14^,^15^). The dominance of maternal only care in mammals is typically linked to sex differences in relative parental investment^12^. It is argued that because males invest less initially, due to anisogamy, they have a higher potential reproductive rate and benefit more from mating multiply than do females^16^. Consequently, selection favored heavy investment in parental care in females and mate seeking and competitive behaviors in males. Therefore, higher optimal mating rates for males render monogamy and care by men an evolutionary puzzle.

Long-standing claims in the literature argue that during human evolution the increased need for paternal investment created selective pressure for long-term pair bonds and a sexual division of labor^17^,^18^. However, growing evidence suggests that paternal care evolves only *after* monogamy becomes established in a population^19^. Because male investment likely would have resulted in male absence (e.g., through resource provisioning), caring males would have faced potential fitness costs due to freerider males that ‘steal’ paternity^5^,^6^. Specifically, males that do not care benefit directly from cuckolded caring males’ investments in offspring that are not theirs. As a consequence, assumptions of paternal care driving monogamy are likely overly simplistic^9^,^20^. For example, a recent survey found that over 40% of socially monogamous species exhibit no indication of male care^21^. Accordingly, while paternal investment may be common across human societies, current patterns should not be confused with the reason that pairing evolved in the first place. Consequently, the question becomes, why did men originally pairbond?

### Why do men pairbond?

Two common alternative arguments for the evolution of monogamy exist in the literature. The first argument suggests that monogamy evolved due to selective pressure favoring males that protected their offspring from attacks by infanticidal competitors^22^,^23^. However, recent phylogenetic analyses cast doubt on this claim and find that across animal taxa the evolution of monogamy is unassociated with the risk of infanticide^21^. The second argument focuses on patterns of female distribution^24^. Solitary females spread across a landscape, due to resource dispersal and/or female intolerance, limit multiple mate monopolization opportunities for males and favor monogamy as a consequence^21^,^25^,^26^. While this argument may hold for many mammals, this explanation is incongruous for many group-living species, including humans and other primates^27^.

A growing body of theoretical and empirical research highlights another pathway to monogamy, and a possible crucial step in the evolution of paternal care, through male mate guarding (defined as the close association between a male and female prior to and/or after copulation for paternity assurance^19^). While research on the evolution of reproductive strategies in humans often reports that the optimal male mating strategy is the pursuit of multiple partners, these modeling approaches typically assign fitness payoffs based on the effort males devote to a particular strategy rather than on the availability of partners^5^,^6^. Consequently, there may be overlooked conditions under which, instead of mating multiply, it may be in the best interest of a male to achieve high paternity with a single female^28^. This trade-off is particularly acute in response to partner availability. When the mating pool is male-biased, males face difficulty in finding additional mates and a current partner becomes a valued resource, favoring mate guarding^8^,^9^,^29^. Accordingly, the adult sex ratio (number of sexually mature males to females in a population; ASR) becomes a key determinant to fitness payoffs of a particular male strategy^7^.

Following recent theoretical and empirical findings, we seek to examine a largely overlooked and possible intermediate step in the evolution of humans from an ancestral multi-male/female mating system with promiscuous mating^1^ to monogamy and paternal care: male mate guarding in response to partner scarcity. We model the response of three male strategies – multiple mate seeking, mate guarding, and paternal care – to fluctuating paternity certainty, benefits to paternal care, and partner availability. In doing so, we seek to offer insight and evaluate key claims for the emergence of monogamy and paternal care in humans.

## Model Specification

### Verbal description of the model

We investigate the selective advantage of three male strategies: Multiple Mating (MM), Mate Guarding (MG), and Paternal Care (PC). Male reproductive success depends on the ratio of available males to females at a particular time and the frequency of the three male strategies. In the model, all males stay in the mating pool during their lifetime except for MG males who leave the mating pool with an encountered female. The probability that a male mates with a female at a particular time is dependent on the ASR, upper-bounded by 1 (i.e., all males will mate when females are in excess), with some variation across the three male strategies.

PC males provide a survival benefit (***c***) to offspring through provisioning. They pair with a female but do not mate guard, therefore there is a probability of cuckoldry (***k***). MM males will attempt to mate with multiple females, with reproductive benefits reflecting shared paternity across available females. If there are PC males in the population, MM males may gain additional reproductive benefits through cuckoldry. MG males, if they meet a female, will guard their partner to prevent cuckoldry, forgoing other mating opportunities^9^,^30^, and mate with the same female during their lifetime, which is determined by the probability of male survival (**u**).

**Figure 1:**
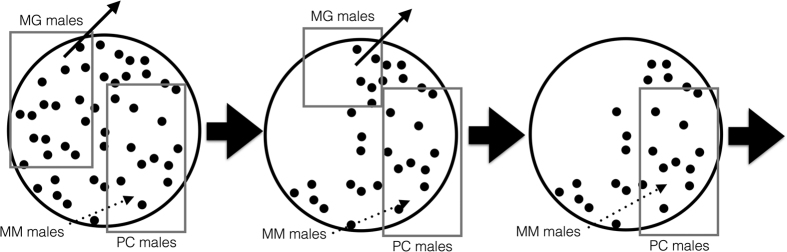
Schematic of the model with all strategies represented. Squares encompass females paired with either Mate Guarding (MG) or Parental Care (PC) males, with MG-paired females leaving the mating pool each time step. Females not in squares have paternity shared among Multiple Mating (MM) males. The dotted arrow represents some paternity given to MM males through cuckoldry.

These dynamics are represented in schematic form in Fig. 1. In step 1, males encounter females randomly, irrespective of their strategy, where the probability of a male encountering a female is solely dependent on the ASR. After an encounter, PC and MG males form pairbonds with females. MM males share paternity across females not paired with MG males and may additionally mate with females paired with PC males, who are at risk to cuckoldry. This risk is determined by three key factors: 1) the willingness of females to engage in extra-pair mating, 2) the possibility that females have not yet become pregnant by a PC male, which is determined by a conception rate parameter in the model (***b***; set to 0.3 following data on monthly conception rates among women without fertility problems^31^) and 3) the frequency of MM males (i.e., if they are rare the probability that they encounter a female paired with a PC male is low). Moving to step 2, MG males paired in step 1 have effectively removed their partners from the mating pool, thereby reducing the number of available females, and the process begins again. Over multiple steps the summed fitness benefit for each strategy reaches an asymptote, which informs the evolutionary dynamics of the three male strategies.

## Results

**Figure 2:**
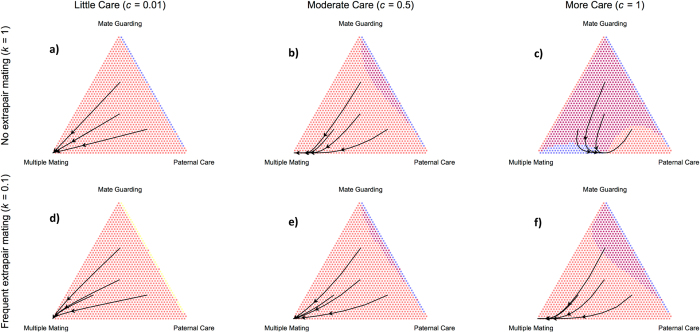
Evolutionary dynamics of the three male mating strategies under a female-biased sex ratio. The panels present six conditions with varying cuckoldry risk and payoffs to parental care (**a–f**) where conception rate (**b**) is set to 0.3. Colors denote which strategy is favored at a given strategy frequency, with yellow indicating Mate Guarding (MG), red Multiple Mating (MM), and blue Paternal Care (PC). Arrows simulate evolutionary trajectories at contrasting mixed-strategy frequencies. Color combination purple indicates when both MM and PC strategies are simultaneously increasing in frequency.

We begin by presenting the evolutionary outcomes due to selection of the three male strategies in response to a mammalian typical female-biased ASR and adult survival probability (**u** = 0.9; see ref. 32), as well as human conception rates (***b*** = 0.3; Fig. 2). With weak and moderate benefits to care, a 1% and 50% increase in offspring survival respectively, we find that MM males are strongly favored regardless of paternity certainty (Fig. 2a,b,d,e). However, when we maximize benefits to care, the level of cuckoldry plays an important role (Fig. 2c,f). Doubling the benefits to care (***c*** = 1) and ensuring paternity certainty yields a mixed strategy equilibrium of PC and MM males (Fig. 2c). Low paternity certainty, however, returns our previous findings favoring MM males (Fig. 2f). In sum, under most conditions, males that pairbond have depressed genetic fitness outcomes relative to MM males due to their restricted sexual strategy in the face of partner abundance. However, if we assume female faithfulness to a heavily investing male^6^, PC males are expected to be as abundant as MM males (Fig. 2c).

**Figure 3:**
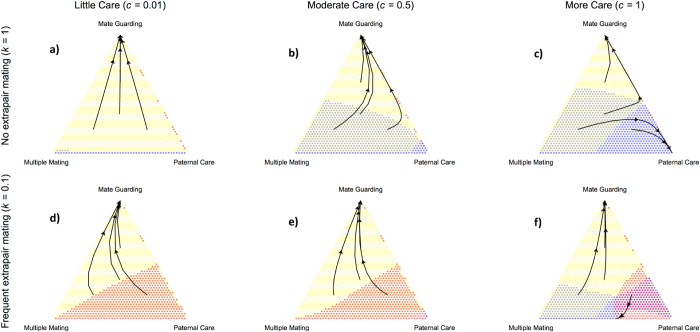
Evolutionary dynamics of the three male mating strategies under a male-biased sex ratio. The panels present six conditions with varying cuckoldry risk and payoffs to parental care (**a–f**) where conception rate (**b**) is set to 0.3. Colors denote which strategy is favored at a given strategy frequency, with yellow indicating Mate Guarding (MG), red Multiple Mating (MM), and blue Paternal Care (PC). Arrows simulate evolutionary trajectories at contrasting mixed-strategy frequencies. Color combination yellow-blue indicates when both MG and PC strategies are simultaneously increasing in frequency.

Next, we move to a male-biased ASR to examine the relative payoffs of the three strategies in response to partner scarcity (Fig. 3). Here, with weak and moderate benefits to care, and regardless of cuckoldry level, we find that MG males are most successful (Fig. 3a,b,d,e). However, as shown above, we again see variation emerge in the optimal male strategy when we double the benefits to care. Instead of a mixed-strategy, as in the female-biased condition, we find that both MG and PC males represent evolutionary stable strategies when paternity certainty is high (Fig. 3c). However, the domain of attraction for PC males decreases with lower paternity certainty, though a mixed strategy between MM and PC males will remain unless a certain threshold of MG males are present (Fig. 3f; see SI Fig. 2 for the role of conception rates that are lower and higher than 0.3 on male strategies). In sum, with low and moderate benefits to care, MG males perform best. However, with maximal benefits to care and no cuckoldry, the PC and MG strategies become competing equilibria^33^.

**Figure 4:**
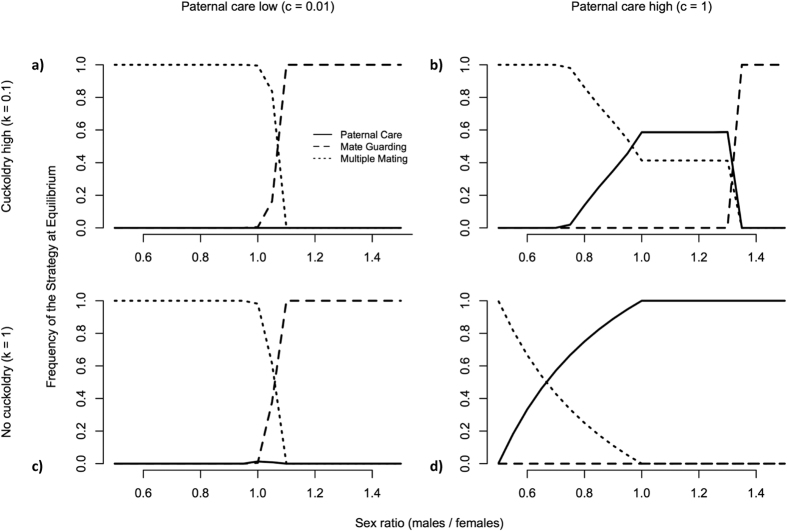
Effects of ASR on equilibrium frequencies of Parental Care (PC), Mate Guarding (MG), and Multiple Mating (MM) strategies when MM is initially common. The four panels (**a–d**) present four conditions with varying levels of parental care and cuckoldry rates. In all simulations the MM strategy is initially common at frequency 0.99, with MG and PC strategies at 0.005. Other parameter values are *b* = 0.3 and *u* = 0.9. See model description for details.

Lastly, we evaluate male fitness outcomes across a range of ASR values (Fig. 4). In particular, we seek to present the relative payoffs for the three strategies when the ASR is at or near parity (1 male for every 1 female) and MM males are initially common (following the typically assumed ancestral condition), while adjusting the benefits to care (c) and rate of cuckoldry (k). When returns to caring are low and cuckoldry common (Fig. 4a), we find that MM is the favored strategy at female-biased ASRs. However, moving right along the x-axis, as the ASR approaches parity, and continues to becomes increasingly male-biased, we see a mixed ESS of MM and MG males, with the equilibrium frequency of MG males increasing until it becomes the (pure) ESS at an ASR of ~1.1 (i.e., 110 males for every 100 females). The story is very similar if we keep payoffs to care low, but remove the risk of cuckoldry (Fig. 4c). While we find some increase in the frequency of PC males when MG males become more common, MG males become the favored strategy more quickly under these conditions. However, when we maximize payoffs to care, we find a mixed equilibrium between PC and MM males across a wider range of ASR values (Fig. 4b). Nonetheless, in male-biased populations (~1.3+), MG males again become most common. This scope for MG males disappears however when we remove cuckoldry risk (Fig. 4d). In sum, we show that under conservative conditions (low benefits to care and high rates of cuckoldry), MG males perform best across male-biased sex ratios. However, under opposite conditions (high benefits to care and low rates of cuckoldry) PC male perform best under a wide range of ASR values (please visit the link https://abell.shinyapps.io/SexRatioSimulation/ to construct your own version of the model to see how varying parameters beyond what we present here affect the relative frequencies of the three strategies and see the Supplementary Information for the R code used to build our models).

## Discussion

Here we are interested in exploring possible pathways for the evolution of monogamy and paternal care in our lineage. To make this question tractable, we allow males to engage in one of three strategies. This is a limitation of our modeling approach, however, with a polymorphic model specification, we can interpret the evolutionary dynamics more clearly in terms of the characteristics of each strategy. Accordingly, we show that when partners are abundant, multiple mating, and not pairbonded, males generally see the greatest fitness returns to their strategy. On the other hand, when males are abundant and partners are rare, males that pairbond generally do best. While we do find scope for the evolution of care in our models, the parameter estimates require paternal care to increase offspring survival considerably in the presence of limited cuckoldry (Figs 2c and 3c). Therefore we believe our findings support previous work that challenges straightforward arguments of a promiscuity to paternal care transition in human evolution. Instead, we offer mate guarding as a possible pathway to elevate paternity certainty and allow for monogamy to evolve in humans. Once pairbonding becomes established, this then allows selection to operate on variation in the amount of care provided by males.

Below we discuss the applications of our findings to: (i) current criticisms of the classical model of sexual selection, (ii) recent research on frequency dependent reproductive decision making, (iii) the evolution of human life history and its sex ratio consequences, and (iv) interpretations for the evolution of paternal care in humans.

Classical sexual selection theory predicts that the relative parental investment of the sexes leads to sex differences in optimal mating rates^12^,^16^,^34^. It is argued that because males invest less in any one reproductive event, as a consequence of anisogamy, they benefit more from mating multiply than do females. Moreover, a persistent theme in the literature is the claim that a shortage of females results in elevated mating effort among males^34^. However, our results do not support claims that male benefits to mating multiply are always high and highlight the importance of frequency dependent dynamics patterning reproductive behavior. We show that it is often in the best interest of a male to forgo pursuing multiple mating opportunities and instead achieve high paternity certainty with a single partner.

These results are in line with a growing body of theoretical and empirical work in the biological and social sciences showing reproductive decisions in response to partner availability that counter conventional assumptions^7^,^36^. For example, among humans, an abundance of men is associated with higher rates of relationship commitment^35^, monogamy^38^,^39^, lower reproductive skew among males^40^, and less promiscuity in both sexes^40^,^42^. These findings are consistent with a recent analysis of 187 bird species^41^ and other studies across diverse animal taxa showing that male-biased sex ratios are consistently associated with higher rates of pairbonding^44^,^45^,^46^,^47^,^48^. Thus, a growing body of literature highlights that partner rarity intensifies male commitment to pairbonded strategies, rather than multiple mate-seeking, across populations of both human and nonhuman animals.

When modeling the evolution of monogamy, we begin by assuming a typical mammalian female-biased ASR^49^. In line with patterns of mammalian mating, we find that across most conditions the optimal strategy for males is to pursue multiple mates. Males that attempt to pairbond do not take advantage of the relative female abundance and as a consequence cannot compete with MM males. However, when we examine male strategies in response to a male-biased ASR, monogamous males perform best. Our findings support empirical research, primarily among birds, showing that where male-biased sex ratios dominate, monogamy is most common^43^,^50^. The relevance of these typically bird-like male-biased sex ratios to human evolution is currently an open question. However, while our closest relatives have typical mammalian female-biased sex ratios^51^, human sex ratios are considerably more male-biased^8^,^36^,^52^ and bird-like^49^,^53^. Thus the question becomes, did our ancestors experience a change in the direction of the sex ratio bias, resulting in a shortage of women? This is a possibility. Because of menopause and our exceptionally long lives, the sex ratio of reproductive-aged individuals in humans is generally male-biased^52^. Therefore, as a consequence of the evolution of increasing longevity, coupled with reproductive cessation in women^54^, ancestral males likely faced an increasingly male-biased sex ratio^8^, altering the selective arena for payoffs to mating strategies.

Our findings also speak to the current debate regarding the relative emergence of monogamy and cooperative breeding in human evolution^54^. The monogamy hypothesis suggests that pairbonding and male care preceded the emergence of cooperative breeding in our lineage^55^. Thus, much of our unique life history can be attributed to male investment. However, cooperative breeding proponents argue that the assistance of others (not the father) increased the fertility of mothers, decreased the mortality of offspring, and allowed for the suite of human life-history traits to evolve^3^,^56^,^57^. While we do not engage this debate directly, we find monogamy in response to partner scarcity. If female scarcity in humans arose primarily due to the extension of the lifespan, then cooperative breeding likely preceded monogamy in humans.

Lastly, for paternal care to emerge in our models, the benefits to care need to be high (e.g., a doubling of offspring survivorship). There are a couple of reasons to doubt how biologically appropriate this parameter estimate is. First, until paternal care has been under selection, it is likely to be inefficient, quite variable in returns, unreliable and possibly of little benefit^58^. Therefore, requiring such high payoffs for care to emerge raises concerns about its relevance to the evolution of monogamy. Second, in a recent cross-cultural review looking to paternal effects on child survival, only a third of studies found any beneficial outcome to father presence^59^. This is obviously quite surprising given how human pairbonds are typically described as a cooperative system of joint production to meet household needs^2^,^17^. With this example we do not seek to challenge whether or not males invest (e.g., paternal care may have benefits other than increasing offspring survival, such as elevating quality^60^) but use it to simply highlight the requirements for paternal care to evolve in our models.

Accordingly, in humans, we contend that the transition from males mating multiply to providing paternal care possibly passed through an intermediate step of male mate guarding in response to partner rarity. This interpretation is consistent with recent phylogenetic analyses of primate social organization, indicating that bonded relationships (i.e., pair-living) derived from an earlier state of multi-male/multi-female groups^61^,^62^. Pairbond formation through mate guarding provides a mechanism to ensure paternity certainty and a possible avenue to open up paternal care to selection. Once pairbond duration lengthens, the reproductive interests of males and females may become aligned. As a derived trait, monogamy may be stabilized through payoffs to infanticide protection^23^ as well as by increasing the interdependence of pairbonded individuals and evolved social mechanisms to maintain the sexual division of labor and the specialization of care tasks^63^. Upon considering the competing equilibria of mate-guarding and parental-care (Fig. 3c), one intriguing possibility is that once PC populations with elevated female fertility and greater degrees of social cooperation emerge, these strategies may become favored and increase in frequency through multilevel selection^32^.

In conclusion, as members of the hominin line began living longer, a transition from a female- to male-bias in the sex ratio was a likely outcome. In response to male abundance, following our model results, mate guarding by males was favored. As males consistently pairbonded and paternity certainty was assured, it became possible for selection to operate on variation across males in the amount of paternal care offered.

## Methods

### Mathematical description of the model

As mentioned above, here we investigate the evolution of three male strategies: PC, MG, and MM, with respective frequencies ***p**, **q***, and 1 −***p*** −***q***. The mating success of each male will depend on the ratio of males and females in the mating pool at time ***t***, (***M***_***t***_**/*****F***_***t***_), and the frequency of the three strategies in a large population. Our approach diverges from previous work on this topic, particularly that mating opportunities are dependent on partner availability.

The reproductive payoffs are structured such that each male enters the mating pool multiple times during its lifetime (Fig. 1), with future benefits discounted by the probability of survival. The sum of these benefits is the fitness of each strategy.

#### Paternal Care strategy

For each time period, PC males will pair with a female with probability ***y***_***t***_** = min [*****F***_***t***_***/M***_***t***_**, 1]** and give an added survival benefit to offspring, ***c***. Since PC males do not guard there is a possibility of cuckoldry by MM males. If ***u*** is the probability of male survival from one period to the next, the fitness of the PC strategy becomes,


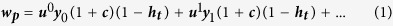


where ***h***_***t***_ is the probability of the PC male providing care to offspring not his own. The expression simplifies to,


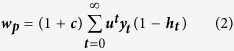


To find ***h***_***t***_, the probability of cuckoldry, we account for the relative frequency of PC and MM males (***p’*** and (1**−*****p’***), respectively), the probability of conception per mating bout (***b***), the willingness of females to engage in extra-pair mating (***k***), and the probability of a female encountering another PC or MM male (***a***_***t***_). In the Supporting Information we show ***h***_***t***_ to be


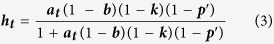


#### Multiple Mating Strategy

MM males attempt to mate with multiple females in each time period, such that the fitness benefit ***z***_***t***_ is gained through shared paternity across available females not being guarded or paired to a PC male. Further, if there are PC males in the population, then cuckoldry will provide added benefits to the MM strategy with ***g***_***t***_ being the expected number of cuckold events per MM male. The fitness of the MM strategy becomes,


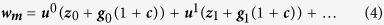


simplifying to


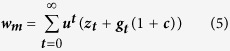


Since the number of females paired with a PC male is ***y***_***t***_***p***_***t***_***M***_***t***_ the maximum possible paternity that an MM male may gain through cuckoldry is,


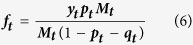


and adjusting for the probability a PC male becomes a cuckold yields,


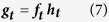


Since the number of females paired to PC and MG males is ***y***_***t***_***p***_***t***_***M***_***t***_ and ***y***_***t***_***q***_***t***_***M***_***t,***_ the number of females for which paternity if shared among MM males is **F**_**t**_−**y**_**t**_**M**_**t**_**(p**_**t**_** + q**_**t**_). Thus,


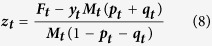


#### Mate Guarding strategy

Following previous theoretical work on the topic^9^ and empirical results linking paternity certainty to time spend guarding^30^, fitness benefits to MG males depend solely on the probability of finding one female, ***y***_***t***_, after which the male guards the female successfully through his lifetime:


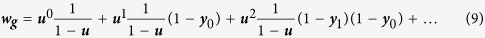


Above simplifies to


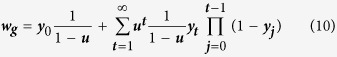


When simulating the fitness of all strategies as they accumulate through time (Fig. 1), they approach an asymptote well before ***t*** = 100. Therefore in all simulations below, we calculate fitness up to this time point.

#### Adult Sex Ratio dynamics

We assume a discrete time process ***t*** = [0, 1, … ] where the fraction of available males to females may change throughout a male’s lifetime. It will change if there are MG males in the population, otherwise it will remain the same as initially determined in each time period. Thus without a significant number of Mate Guarding males (**q** ≈ 0) then ***M***_***t***_ = ***M***_***0***_ and ***F***_***t***_ = ***F***_***0***_, and the fitness of the strategies specified above simplifies greatly. If there are MG males in the population then ***M***_***t***_ and ***F***_***t***_ will change through time as females encountering MG males leave the mating pool. Once MG males are fully gone, then the sex ratio of available females to males is constant. Importantly, the effects MG males have on the operational sex ratio (i.e., those available to mate; OSR) varies both with the ASR and their frequency. In a male-biased ASR, when mate guarding males are common, they reduce the numbers of available females to near 0. However, the effect MG males have on the OSR is much different at female-biased ASRs. These males, by removing themselves from the population, increase the relative numbers of females available to, for example, MM males (see SI).

If there are ***F***_***t***_ females and ***q***_***t***_***M***_***t***_ MG males in the population and ***M***_***t***_ > ***F***_***t***_, then the probability of a female becoming newly guarded by a male is ***q***_***t***_, making the average number of females being newly guarded by a male is ***q***_***t***_***F***_***t***_. With ***M***_***t***_ and ***F***_***t***_ being the number of males and females in the mating pool at time ***t***, and ***q***_***t***_ the frequency of non-paired MG males, then


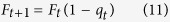



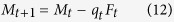



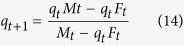


In Figs 2 and 3 we investigate contrasting initial sex ratios that are female-biased (***M***_***0***_ = 100*, **F***_***0***_ = 150) and male-biased (***M***_***0***_ = 150*, **F***_***0***_ = 100). In Fig. 4 we vary the ASR along a continuous scale.

## Additional Information

**How to cite this article**: Schacht, R. and Bell, A. V. The evolution of monogamy in response to partner scarcity. *Sci. Rep.* **6**, 32472; doi: 10.1038/srep32472 (2016).

## Supplementary Material

Supplementary Information
