# Supplementary material for: The evolution of monogamy in response to partner scarcity
Source: Sci Rep. 2016 Sep 7;6:32472. doi: 10.1038/srep32472 (PMC5013280; doi:10.1038/srep32472)
Supplement: Supplementary Information [file srep32472-s1.pdf]

SUPPLEMENTARY INFORMATION
The evolution of monogamy in response to partner scarcity

Adrian V. Bell and Ryan Schacht

Department of Anthropology, University of Utah
270 S. 1400 E. Rm102, SLC, UT 84112

July 15, 2016

Deriving the probability of cuckoldry

To find h_t, the probability of cuckoldry mentioned in the main text, we account for the relative frequency of PC males to MM males, noted p'_t and $(1 - p'_t)$ respectively. This is because MG males do not engage in extra pair matings and MM males do not have access to the females paired with MG males. For a PC male we can represent the time spent caring for their own offspring or another male's as a Markov process using a transition probability matrix:

	Mate	Care	Cuckoldry
Mate	$(1-b)((1-k)(1-a(1-p')b)+k)$	b	$(1-b)(1-k)a(1-p')b$
Care	$1-\alpha$	α	0
Cuckoldry	$1-\alpha$	0	α

Transition probability matrix describing the probability of a PC male caring for his own offspring or the offspring of another male. The state *Mate* represents time spent in mating bouts with a female that is not pregnant, *Care* represents the time spent providing care for a PC male's own offspring, and *Cuckoldry* represents the time spent providing care for a MM male's offspring. Transitioning from *Mate* to *Care* is simply a function of the probability of conception per mating bout b, and transitioning from *Mate* to *Mate* or *Mate* to *Cuckoldry* depends on the probability of extra-pair conception from available males.

In the transition matrix, b is the probability of conception per mating bout[1], k is the level of social control by PC males to prevent cuckoldry (e.g. female choice), and $a_t = \min\{M_t/F_t, 1\}$ is the probability of a female encountering another PC or MM male. We can solve the expected time

[1] see reference X in the main text showing the narrow time windows where conception can occur, thus b can be fairly low

spent in each state and simplify further assuming $\alpha \to 1$, yielding,

$$E\left[\text{Mate}\right] = 0$$

$$E\left[\text{Care}\right] = \frac{1}{1 + a(1-b)(1-k)(1-p')}$$

$$E\left[\text{Cuckoldry}\right] = \frac{a(1-b)(1-k)(1-p')}{1 + a(1-b)(1-k)(1-p')}$$

Since we assume $\alpha \to 1$, *Care* and *Cuckoldry* become absorbing states. We can thus equate the result for $E\left[\text{Cuckoldry}\right]$ to the probability of being a cuckold, h_t. In the simulations to follow, we define a function `pr.cuckold` to define this probability,

```
pr.cuckold <- function(  a, b, k, p ){
a*(1-b)*(1-k)*(1-p)/(1+a*(1-b)*(1-k)*(1-p))
}
```

where you can see the effects of the different parameters above.

```
pr.cuckold(  a=1, b=0.6, p=0.5, k=0.6 )
sapply( seq(0,1,0.01), function(z) pr.cuckold(  a=1, b=0.5, p=0.5, k=z ) )
```

Operational Sex Ratio dynamics

In the main text we specified recursions describing how the operational sex ratio changes through time as MG males (if present) effectively take females out of the mating pool. In the figure below we simulate these recursions to demonstrate the effect of MG males on sex ratios across time steps:

As expected, more MG males results in the sex ratio changing significantly as females are effectively taken out of the mating pool. The direction of change, however, depends on whether the mating pool begins male-biased or female-biased. Male-biased sex ratios with many MG males results in a very low ratio of females to males, while female-biased sex ratios with many MG males results in a much higher ratio of females per male.

To get the graph above, we use the system described in the main text to write a function that takes initial numbers of females and males of different mating strategies. First lets define a function `asr.sim` which simulates the number of females and males in the mating pool through time. It also keeps track of the frequency of MG males in the population.

```
asr.sim <- function( f, m, q, t ){
ft = mt = qt = rep(NA, t)
if( m>f ){
ft[1]=f; mt[1]=m; qt[1]=q
for( i in 2:t ){
ft[i]<-ft[i-1]*(1-qt[i-1])
mt[i]<-mt[i-1] - qt[i-1]*ft[i-1]
qt[i]<-( qt[i-1]*mt[i-1]-qt[i-1]*ft[i-1] )/(mt[i-1]-qt[i-1]*ft[i-1])
```

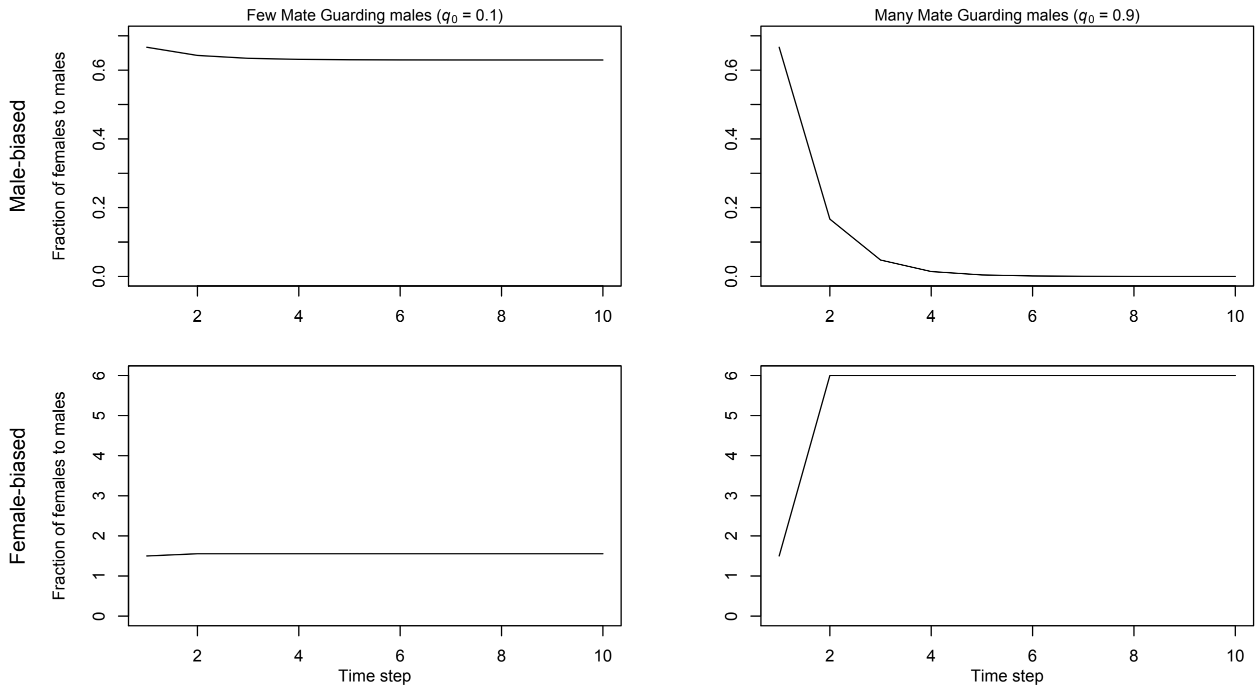

Supp. Fig. 1: Changes in the operational sex ratio due to Mate Guarding males of low (left column) and high frequencies (right column). The top and bottom row have male-biased and female-biased sex ratios, respectively.

```
}
}
if( f>=m ){
ft[1]=f; mt[1]=m; qt[1]=q
ft[2:t] <- f - q*m
mt[2:t] <- mt[1]*( 1 - qt[1] )
qt[2:t] <- 0
}
ans<-list( ft,mt,qt )
names( ans ) <- c("ft", "mt", "qt")
ans
}
```

This function returns the number of females, males, and frequency of MG males until time t. Using this function we can create the figure above using the code below. You may wish to change the parameters a bit.

```
par( mfrow=c(2,2), mar=c(3,6,1,1), cex=0.8 )
time = 10
par.sp1 <- asr.sim( f=100, m=150, q=0.1, t=time )
plot( 1:length(par.sp1$ft), par.sp1$ft/par.sp1$mt, type="l",
ylab="Fraction of females to males", xlab="",
main=expression( paste( "Few Mate Guarding males (",italic(q[0])," = 0.1)")),
```

```
ylim=c(0,0.7), cex.main=0.95 )
mtext( "Male-biased", side=2, line=5 )
par.sp1 <- asr.sim( f=100, m=150, q=0.9, t=time )
plot( 1:length(par.sp1$ft), par.sp1$ft/par.sp1$mt, type="l", ylab="", xlab="",
main=expression( paste( "Many Mate Guarding males (",italic(q[0])," = 0.9)")),
ylim=c(0,0.7), cex.main=0.95)

par.sp1 <- asr.sim( f=150, m=100, q=0.1, t=time )
plot( 1:length(par.sp1$ft), par.sp1$ft/par.sp1$mt, type="l",
ylab="Fraction of females to males", xlab="", ylim=c(0,6) )
mtext( "Time step", side=1, line=2, cex=0.8)
mtext( "Female-biased", side=2, line=5 )
par.sp1 <- asr.sim( f=150, m=100, q=0.9, t=time )
plot( 1:length(par.sp1$ft), par.sp1$ft/par.sp1$mt, type="l", ylab="", xlab="", main="", ylim=c(0,6))
mtext( "Time step", side=1, line=2, cex=0.8)
```

Evolutionary dynamics

We use a standard viability recursion to simulate the change in the frequency of a strategy due to selection.

$$p_{t+1} = p_t w_p / \bar{w} \quad (1)$$
$$q_{t+1} = q_t w_g / \bar{w} \quad (2)$$

where $\bar{w} = p_t w_p + q_t w_g + (1 - p_t - q_t) w_m$. To simulate evolutionary dynamics we first define the function `fitness` measuring the fitness of PC, MM, and MG males. This follows directly from the specification in the main text.

```
# fitness of strategies
fitness <- function( p0, q0, t=100, c, u, b, f0, m0, k ){
 # first find how the operational sex ratio changes through time
 asr.dyn<-asr.sim( f=f0, m=m0, q=q0, t=t )
 # reassign variables
 ft <- asr.dyn$ft; mt <- asr.dyn$mt; qt <- asr.dyn$qt
 # define vector for the frequency of PC over time
 pt <- rep(NA, t); pt[1] <- p0
for( i in 2:t ) pt[i] <- pt[i-1] + ( qt[i-1] - qt[i] )/2

 # define variables representing reproductive
 # payoffs each time period
 # for PC, MM, and MG males
 vp <- ve <- vg <- 0
 # loop over time periods
 for( i in 1:t ){
 if( mt[i]>0 ){ # if there are males left
 # PC and MM relative frequencies (not counting MG males)
 pc <- ifelse( qt[i]==1, 0, pt[i]/( 1 - qt[i] ) )

 # prob of a female encountering a male after
 # MG males have paired with females, set ceiling at one
 # and condition for the last time step
 apc <- min( 1, ifelse( i==t, mt[i]/ft[i], mt[i+1]*(1-qt[i+1]) / ft[i+1] ) )
 # checks to make sure it is a valid value
 apc <- ifelse( is.nan(apc), 0, apc)
 apc <- ifelse( is.infinite(apc), 0, apc)

 # now define variable y, defined in the main text
 yt <- min( 1, ft[i]/mt[i] )

 # calculate reproductive payoff at time i, following the main text

 # parental care (vp)
 vp <- vp + (1+c) * u^(i-1) * min( ft[i]/mt[i], 1) * ( 1 - pr.cuckold( a=apc, b=b, p=pc, k=k ) )

 # multiple mating (ve)
 f <- ifelse( (1 - pt[i] - qt[i]) > 0, yt * pt[i] / ( 1 - pt[i] - qt[i] ), 0 )
 gt <- f * pr.cuckold( a=apc, b=b, p=pc, k=k )
```

```
 zt <- ifelse( (1 - pt[i] - qt[i]) > 0, ( ft[i] - yt * mt[i] * (pt[i]+qt[i]) ) /
 ( mt[i] * (1 - pt[i] - qt[i] ) ), 0 )

 ve <- ve + u^(i-1) * ( zt + gt*(1+c) )

 # mate guarding (vg)
 fnot <- ifelse( i>1, prod( sapply( seq(1,i-1,1), function(z) 1 -  min( ft[z]/mt[z], 1) ) ), 1 )
 vg <- vg + u^(i-1) * min( ft[i]/mt[i], 1) * 1/(1-u) * fnot
}
}
 # return final fitness values
 ans<-c( vp, vg, ve )
 names(ans) <- c("Wp", "Wg", "We")
 ans
 }
```

We can test the output by the following call:

```
fitness( p0=0, q0=1, t=10, c=0.2, u=0.9, b=0.25, f0=100, m0=100, k=0.2)
```

Simulation of stable equilibria with changing sex ratio

Figure 4 in the main text illustrates the equilibrium frequency of PC, MM, and MG strategies given an initial sex ratio. Below we provide the code for this figure. First we define a function finding the change in frequency of each strategy given a set of parameter values.

```
delta.sim <- function( startx, starty, c=c, u=u, b=b, f0=f0, m0=m0, k=k ) {
  p <- startx
  q <- starty
  ## call to get fitness values
  fitpq <- fitness(p0=p, q0=q, t=100, c=c, u=u, b=b, f0=f0, m0=m0, k=k)

  fit <- c( fitpq["Wp"], fitpq["Wg"], fitpq["We"] ) # return fitnesses
  wbar <- p*fit[1] + q*fit[2] + (1-p-q)*fit[3];
  # change in PC frequency
  dp <- p*fit[1]/wbar - p;
  # change in MG frequency
  dq <- q*fit[2]/wbar - q;
  # change in MM frequency
  dr <- (1-p-q)*fit[3]/wbar - (1-p-q);

  # ensure the change in freq. adds up to 0
  con <- ( dp + dq + dr )/3
  dp <- ifelse( dp==0, 0, dp - con )
  dq <- ifelse( dq==0, 0, dq - con )
  dr <- ifelse( dr==0, 0, dr - con )

 # return frequency changes
  c(dp,dq,dr)
}
```

Now using the above function `delta.sim`, we accumulate the frequency changes to track the evolutionary dynamics:

```
sim.pq <- function( p0, q0, time, c, u, b, f0, m0, k ){
pt <- qt <- rep(NA,time)
pt[1] <- p0
qt[1] <- q0
for( i in 2:time ){
      deltapq <- delta.sim( pt[i-1], qt[i-1], c=c, u=u, b=b, f0=f0, m0=m0, k=k )
      pt[i] <- pt[i-1] + deltapq[1]
      qt[i] <- qt[i-1] + deltapq[2]
}
ans <- list(pt,qt); names(ans) <- c("pt","qt")
ans
}
```

We can test this function:

```
sim.pq( p0=0.9, q0=0.05, time=5, c=10, u=0.9, b=0.5, f0=150, m0=100, k=1 )
```

Now we create a function to gather the equilibrium frequencies with varying initial sex ratios and optionally plot them.

```
sr.sim <- function( p0, q0, time=100, c=2, u=0.9, b=0.3, k=1, plotit=FALSE){
# function for plot
mseq <- seq(50,150,5)
fseq <- rep(100,length(mseq) )

eq.strat <- matrix( rep(NA,2*length(mseq)), nrow=length(mseq) )

for( i in 1:length(mseq)){
strat.dyn <- sim.pq( p0=p0, q0=q0, time=time, c=c, u=u, b=b, f0=fseq[i], m0=mseq[i], k=k )
eq.strat[i,] <- c( strat.dyn$pt[time], strat.dyn$qt[time] )
}

ratio <- mseq / fseq
if( plotit==TRUE ){
quartz()
plot( ratio, eq.strat[,1], axes=FALSE, type="n", ylab="", xlab="", ylim=c(0,1) )
axis( side=1)
mtext( "Sex ratio F/M", side=1, line=2)
mtext( "Equilibrium frequencies", side=2, line=2)
axis( side=2)
lines( ratio, eq.strat[,1], lty=1, lwd=2)
lines( ratio, eq.strat[,2], lty=2, lwd=2)
lines( ratio, 1 - eq.strat[,1] - eq.strat[,2], lty=3, lwd=2)
legend( "topright", legend=c("PC","MG", "MM"), lty=1:3)
}
ans <- list(eq.strat, ratio); names(ans) <- c("eq.strat", "ratio")
ans
}
```

Now we use the function `sr.sim` to create Fig. 4 for the paper. We assume below that the MM strategy is initially common.

```
# MM common low care high cuckoldry
mm_com_lh <- sr.sim( p0=0.005, q0=0.005, t=100, c=.01, u=0.9, b=0.3, k=0.1 )
# MM common low care low cuckoldry
mm_com_ll <- sr.sim( p0=0.005, q0=0.005, t=100, c=.01, u=0.9, b=0.3, k=1 )
# MM common high care high cuckoldry
mm_com_hh <- sr.sim( p0=0.005, q0=0.005, t=100, c=1, u=0.9, b=0.3, k=0.1 )
# MM common high care low cuckoldry
mm_com_hl <- sr.sim( p0=0.005, q0=0.005, t=100, c=1, u=0.9, b=0.3, k=1 )

quartz(width=9, height=6)
par(oma=c(3,4,3,1), mar=c(2,4,2,1), mfcol=c(2,2), cex=0.8 )
plot( mm_com_lh$ratio, mm_com_lh$eq.strat[,1], axes=FALSE, type="n", ylab="", xlab="", ylim=c(0,1) )
axis( side=1, at=seq(0.6,2.0,0.2))
mtext( "Frequency of the Strategy at Equilibrium", side=2, line=3, cex=0.8, at=-0.3)
mtext( "Cuckoldry high (k = 0.1)", side=2, line=6, cex=0.8)
mtext( "Parental care low (c = 0.01)", side=3, line=3, cex=0.8 )
axis( side=2)
lines( mm_com_lh$ratio, mm_com_lh$eq.strat[,1], lty=1, lwd=2)
lines( mm_com_lh$ratio, mm_com_lh$eq.strat[,2], lty=2, lwd=2)
lines( mm_com_lh$ratio, 1 - mm_com_lh$eq.strat[,1] - mm_com_lh$eq.strat[,2], lty=3, lwd=2)
legend( "right", legend=c("Parental Care","Mate Guarding", "Multiple Mating"), lty=1:3, box.lwd=0,
cex=0.8)

plot( mm_com_ll$ratio, mm_com_ll$eq.strat[,1], axes=FALSE, type="n", ylab="", xlab="", ylim=c(0,1) )
axis( side=1)
mtext( "Sex ratio (males / females)", side=1,line=3, cex=0.8, at=1.6)
mtext( "No cuckoldry (k = 1)", side=2, line=6, cex=0.8)
mtext( "", side=3 )
axis( side=2)
lines( mm_com_ll$ratio, mm_com_ll$eq.strat[,1], lty=1, lwd=2)
lines( mm_com_ll$ratio, mm_com_ll$eq.strat[,2], lty=2, lwd=2)
lines( mm_com_ll$ratio, 1 - mm_com_ll$eq.strat[,1] - mm_com_ll$eq.strat[,2], lty=3, lwd=2)

plot( mm_com_hh$ratio, mm_com_hh$eq.strat[,1], axes=FALSE, type="n", ylab="", xlab="", ylim=c(0,1) )
axis( side=1)
#mtext( "Sex ratio F/M", side=1, line=3)
#mtext( "", side=2, line=2)
mtext( "Parental care high (c = 1)", side=3, line=3, cex=0.8)
axis( side=2)
lines( mm_com_hh$ratio, mm_com_hh$eq.strat[,1], lty=1, lwd=2)
lines( mm_com_hh$ratio, mm_com_hh$eq.strat[,2], lty=2, lwd=2)
lines( mm_com_hh$ratio, 1 - mm_com_hh$eq.strat[,1] - mm_com_hh$eq.strat[,2], lty=3, lwd=2)

plot( mm_com_hl$ratio, mm_com_hl$eq.strat[,1], axes=FALSE, type="n", ylab="", xlab="", ylim=c(0,1) )
axis( side=1)
#mtext( "Sex ratio Males/Females", side=1, line=3, cex=0.8)
mtext( "", side=2, line=2)
#mtext( "MM common", side=3 )
```

```
axis( side=2)
lines( mm_com_hl$ratio, mm_com_hl$eq.strat[,1], lty=1, lwd=2)
lines( mm_com_hl$ratio, mm_com_hl$eq.strat[,2], lty=2, lwd=2)
lines( mm_com_hl$ratio, 1 - mm_com_hl$eq.strat[,1] - mm_com_hl$eq.strat[,2], lty=3, lwd=2)
```

To produce Supp. Fig. 3, we used the above code but assumed $u = 0$.

Evolutionary dynamics illustrated on ternary plots

Figures 2 and 3 of the main text illustrate the evolutionary dynamics of PC, MM, and MG using ternary plots. The R code for the ternary plots themselves were modified from the original code of a package Baryplot created by Richard McElreath, found here. Our modified code is found here. After loading all of the R functions associated with Baryplot, we used the function `fitness` defined above to create the plots.

```
# define ternary plot function
tern.asr = function(t, c, u, b, f0, m0, k, length=0.012, cont.pqby=0.025, phase.pqby=0.03,
t.contour=F, t.phase=F, t.arrows=F, labcex=0.8 ){
 # game fitness function
  game = function( p, q, w0=1 ){
  ans = fitness(p0=p, q0=q, t=t, c=c, u=u, b=b, f0=f0, m0=m0, k=k)
  c( ans["Wp"], ans["Wg"], ans["We"] ) # return fitnesses
 }
 # run baryplot
   bary.init(col="white")
 bary.labels("Paternal Care","Mate Guarding", "Multiple Mating",cex=labcex)

 if( t.contour==T ) bary.contour2(thegame = game, pqby=cont.pqby )

 # phase plot
 if( t.phase==T ) bary.phase(thegame = game, length = length, pqby=phase.pqby)

 if( t.arrows==T ){
 # around the center
  bary.plotsim( 0.33, 0.33, arrow=TRUE, thegame=game )
  bary.plotsim( 0.2, 0.2, arrow=TRUE, thegame=game )
  bary.plotsim( 0.6, 0.2, arrow=TRUE, thegame=game )
  bary.plotsim( 0.2, 0.6, arrow=TRUE, thegame=game )
   }
 }
```

With the plotting function `tern.asr` defined, we can produce Fig. 2 and 3 of the main text.

```
# FIGURE 2
plotarrows=T
labcex=0.6
titlecex=0.9
quartz( width=11, height=5 )
b.global <- 0.5
```

```
k.high <- 1
k.low <- 0.1

par( fig=c(0,0.33,0.5,1), mar=c(0.55,0,1,0) )
tern.asr(t=100, c=0.01, u=0.9, b=b.global , f0=100, m0=150, k=k.high, t.arrows=plotarrows,
t.contour=T,cont.pqby=0.02,  labcex=labcex) # little care
mtext( expression( paste("Little Care (",italic(c)," = 0.01)")), side=3, cex=titlecex)
mtext( expression( paste( "No extrapair mating (",italic(k)," = 1)")), side=2, line=-1, cex=titlecex)

par( fig=c(0.33,0.66,0.5,1), new=TRUE )
tern.asr(t=100, c=0.5, u=0.9, b=b.global , f0=100, m0=150, k=k.high, t.arrows=plotarrows,
t.contour=T,cont.pqby=0.02, labcex=labcex) # moderate care
mtext( expression( paste( "Moderate Care (",italic(c)," = 0.5)")), side=3, cex=titlecex)

par( fig=c(0.66,1,0.5,1), new=TRUE )
tern.asr(t=100, c=1, u=0.9, b=b.global , f0=100, m0=150, k=k.high, t.arrows=plotarrows,
t.contour=T,cont.pqby=0.02, labcex=labcex) # more care
mtext( expression( paste( "More Care (",italic(c)," = 1)")), cex=titlecex, side=3)

# --------------
par( fig=c(0,0.33,0,0.5), new=TRUE )
tern.asr(t=100, c=0.01, u=0.9, b=b.global , f0=100, m0=150, k=k.low, t.arrows=plotarrows,
t.contour=T, cont.pqby=0.02,  labcex=labcex) # little care
mtext( expression( paste( "Frequent extrapair mating (",italic(k)," = 0.1)")), side=2, line=-1,
cex=titlecex)
par( fig=c(0.33,0.66,0,0.5), new=TRUE )
tern.asr(t=100, c=0.5, u=0.9, b=b.global , f0=100, m0=150, k=k.low, t.arrows=plotarrows,
t.contour=T, cont.pqby=0.02, labcex=labcex) # moderate care
par( fig=c(0.66,1,0,0.5), new=TRUE )
tern.asr(t=100, c=1, u=0.9, b=b.global , f0=100, m0=150, k=k.low, t.arrows=plotarrows,
t.contour=T, cont.pqby=0.02, labcex=labcex) # more care

# FIGURE 3
plotarrows=T
labcex=0.6
titlecex=0.9
quartz( width=11, height=5 )
b.global <- 0.5
k.high <- 1
k.low <- 0.1

par( fig=c(0,0.33,0.5,1), mar=c(0.55,0,1,0) )
tern.asr(t=100, c=0.01, u=0.9, b=b.global , f0=150, m0=100, k=k.high, t.arrows=plotarrows,
t.contour=T,cont.pqby=0.02,  labcex=labcex) # little care
mtext( expression( paste("Little Care (",italic(c)," = 0.01)")), side=3, cex=titlecex)
mtext( expression( paste( "No extrapair mating (",italic(k)," = 1)")), side=2, line=-1, cex=titlecex)

par( fig=c(0.33,0.66,0.5,1), new=TRUE )
tern.asr(t=100, c=0.5, u=0.9, b=b.global , f0=150, m0=100, k=k.high, t.arrows=plotarrows,
t.contour=T,cont.pqby=0.02, labcex=labcex) # moderate care
mtext( expression( paste( "Moderate Care (",italic(c)," = 0.5)")), side=3, cex=titlecex)
```

```
par( fig=c(0.66,1,0.5,1), new=TRUE )
tern.asr(t=100, c=1, u=0.9, b=b.global , f0=150, m0=100, k=k.high, t.arrows=plotarrows,
t.contour=T,cont.pqby=0.02, labcex=labcex) # more care
mtext( expression( paste( "More Care (",italic(c)," = 1)")), cex=titlecex, side=3)

# --------------
par( fig=c(0,0.33,0,0.5), new=TRUE )
tern.asr(t=100, c=0.01, u=0.9, b=b.global , f0=150, m0=100, k=k.low, t.arrows=plotarrows,
t.contour=T, cont.pqby=0.02,  labcex=labcex) # little care
mtext( expression( paste( "Frequent extrapair mating (",italic(k)," = 0.1)")), side=2, line=-1,
cex=titlecex)
par( fig=c(0.33,0.66,0,0.5), new=TRUE )
tern.asr(t=100, c=0.5, u=0.9, b=b.global , f0=150, m0=100, k=k.low, t.arrows=plotarrows,
t.contour=T, cont.pqby=0.02, labcex=labcex) # moderate care
par( fig=c(0.66,1,0,0.5), new=TRUE )
tern.asr(t=100, c=1, u=0.9, b=b.global , f0=150, m0=100, k=k.low, t.arrows=plotarrows,
t.contour=T, cont.pqby=0.02, labcex=labcex) # more care
```

The code to produce Supp. Fig. 2, investigating the level of extra pair mating and conception rate.

```
plotarrows=T
labcex=0.6
titlecex=0.9
quartz( width=11, height=5 )

par( fig=c(0,0.33,0.5,1), mar=c(0.55,0,1,0) )
tern.asr(t=100, c=1, u=0.9, b=0.2 , f0=100, m0=150, k=0.1, t.arrows=plotarrows,
t.contour=T,cont.pqby=0.02,  labcex=labcex) # little care
mtext( expression( paste("Frequent extrapair mating (",italic(k)," = 0.1)")), side=3, cex=titlecex)
mtext( expression( paste( "Moderate conception rate (",italic(b)," = 0.2)")), side=2, line=-1,
cex=titlecex)

par( fig=c(0.33,0.66,0.5,1), new=TRUE )
tern.asr(t=100, c=1, u=0.9, b=0.2 , f0=100, m0=150, k=0.5, t.arrows=plotarrows,
t.contour=T,cont.pqby=0.02, labcex=labcex) # moderate care
mtext( expression( paste( "Moderate extrapair mating (",italic(k)," = 0.5)")), side=3, cex=titlecex)

par( fig=c(0.66,1,0.5,1), new=TRUE )
tern.asr(t=100, c=1, u=0.9, b=0.2 , f0=100, m0=150, k=0.9, t.arrows=plotarrows,
t.contour=T,cont.pqby=0.02, labcex=labcex) # more care
mtext( expression( paste( "Rare extrapair mating (",italic(k)," = 0.9)")), cex=titlecex, side=3)

# conception rate
par( fig=c(0,0.33,0,0.5), mar=c(0.55,0,1,0), new=TRUE )
tern.asr(t=100, c=1, u=0.9, b=0.01 , f0=100, m0=150, k=0.1, t.arrows=plotarrows,
t.contour=T,cont.pqby=0.02,  labcex=labcex) # little care
mtext( expression( paste( "Low conception rate (",italic(b)," = 0.01)")), side=2, line=-1,
cex=titlecex)
```

```
par( fig=c(0.33,0.66,0,0.5), new=TRUE )
tern.asr(t=100, c=1, u=0.9, b=0.01 , f0=100, m0=150, k=0.5, t.arrows=plotarrows,
t.contour=T,cont.pqby=0.02, labcex=labcex) # moderate care

par( fig=c(0.66,1,0,0.5), new=TRUE )
tern.asr(t=100, c=1, u=0.9, b=0.01 , f0=100, m0=150, k=0.9, t.arrows=plotarrows,
t.contour=T,cont.pqby=0.02, labcex=labcex) # more care
```

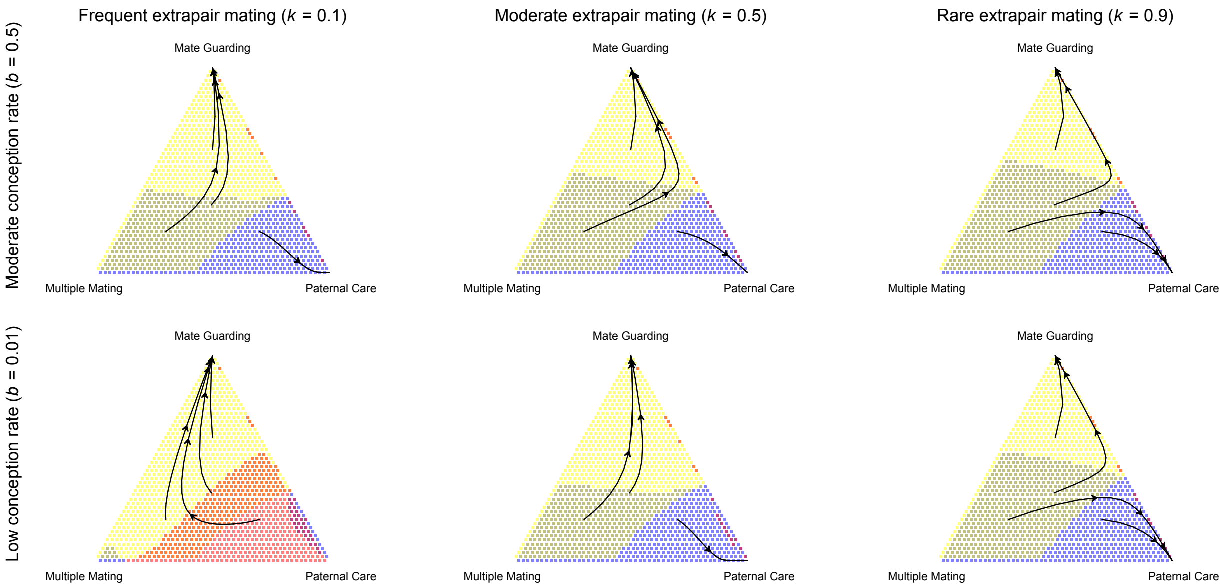

Supp. Fig. 2: Evolutionary dynamics of the three male mating strategies under a male-biased sex ratio when the conception rate (b) and the extrapair mating parameter (k) is varied. Parental care is high at $c = 1$. Colors denote which strategy is favored at a given strategy frequency, with yellow indicating Mate Guarding (MG), red Multiple Mating (MM), and blue Paternal Care (PC). Arrows simulate evolutionary trajectories at contrasting initial frequencies. Color combination brown indicates when both MG and PC strategies are simultaneously increasing in frequency. Moderate increases in the conception rate and the extrapair mating parameter favor the two pure strategy equilibrium of either MG males or PC males.

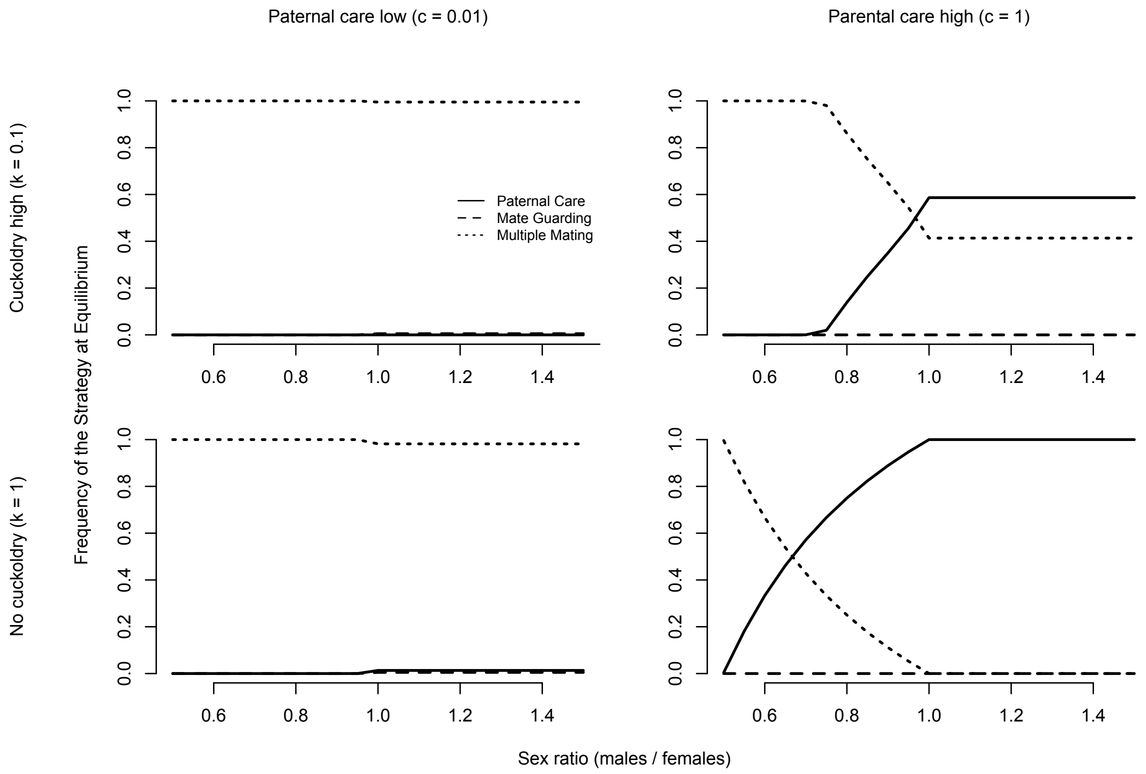

Supp. Fig. 3: Effects of sex ratio on equilibrium frequencies of Parental Care (PC), Mate Guarding (MG), and Multiple Mating (MM) strategies. The four panels present four conditions varying levels of parental care and cuckoldry rates. In all simulations the MM strategy is initially common at frequency 0.99, with MG and PC strategies at 0.005. Other parameter values are $b = 0.3$ and $u = 0$, with the latter parameter value prescribing that this is the special case where there is one mating bout per generation. This assumption heavily disfavors the pairing strategies PC and MG. See model description for details.
